# Transcriptomic and Single-Nucleus Characterization of a Tumor Microenvironment-Associated Four-Gene Score in Esophageal Adenocarcinoma

**DOI:** 10.3390/cimb48101084

**Published:** 2026-10-20

**Authors:** Fang Fu, Chaoxin Gao, Haiyang Chen, Yi Chen

**Affiliations:** 1 West China Centre of Excellence for Pancreatitis and Laboratory of Metabolism and Aging, Frontiers Science Center for Disease-Related Molecular Network and National Clinical Research Center for Geriatrics, West China Hospital, Sichuan University, Chengdu 610041, China; fufang@stu.scu.edu.cn (F.F.); gcxchaoxin@163.com (C.G.); 2 West China School of Medicine, Sichuan University, Chengdu 610041, China; 3 Division of Gastrointestinal Surgery, Department of General Surgery, West China Hospital, Sichuan University, Chengdu 610041, China

**Keywords:** esophageal adenocarcinoma, prognostic signature, tumor immune microenvironment, molecular stratification, nomogram, LASSO–Cox regression

## Abstract

Esophageal adenocarcinoma (EAC) is molecularly heterogeneous, and prognostic models with clear biological context remain limited. We analyzed RNA-sequencing and clinical data from 46 EAC tumors and 13 adjacent normal tissues in The Cancer Genome Atlas. Differential expression, ESTIMATE, weighted gene co-expression network analysis, and LASSO–Cox regression were used to derive a tumor microenvironment-associated score, followed by bootstrap and nested cross-validation, assessment in independent cohorts, immune deconvolution, and single-nucleus RNA-sequencing analysis. We identified 1571 differentially expressed genes and an 879-gene module associated with stromal, immune, and tumor-purity traits. A four-gene score comprising *ACSM1*, *AGT*, *IGF2BP3*, and *ESM1* stratified overall survival in the development cohort. The optimism-corrected C-index was 0.769, whereas the median nested cross-validation C-index was 0.589. In GSE13898, the locked score identified Cluster B with a rank AUC of 0.923; however, survival associations were not reproduced in GSE72873 or GSE254942. Immune-cell comparisons showed nominal differences that did not remain significant after BH correction. Single-nucleus analysis localized *IGF2BP3* mainly to malignant cells and *ESM1* to endothelial cells. The score provides molecular-subtype and cellular context for hypothesis generation, but its prognostic generalizability remains unestablished.

## 1. Introduction

Esophageal cancer remains a major cause of cancer mortality, with an estimated 604,100 new cases and 544,100 deaths worldwide in 2020 [1,2]. Its two predominant histological forms, esophageal adenocarcinoma (EAC) and esophageal squamous cell carcinoma, differ in epidemiology, molecular architecture, and treatment response and should therefore be studied as separate disease entities [1,3]. EAC accounts for a growing proportion of cases in many Western populations, yet its clinical course remains difficult to predict. Conventional stage captures the anatomical extent of disease but cannot fully represent the biological heterogeneity that contributes to differences in progression and survival. Transcriptome-based markers may complement clinicopathological assessment by identifying molecular programs that are not apparent from morphology alone.

EAC is characterized by extensive genomic instability and marked transcriptional diversity [3,4]. Part of this diversity reflects interactions between malignant cells and the surrounding stromal and immune compartments. The composition and functional state of the tumor microenvironment have been associated with treatment response and outcome, whereas immune infiltration alone does not necessarily indicate effective antitumor activity [5,6]. Bulk transcriptomic data can capture these coordinated tumor–microenvironment signals, but their interpretation is complicated by cellular admixture. Recent single-nucleus profiling has further shown that inflammatory and immune-regulatory programs in EAC arise from distinct malignant and non-malignant cell compartments [7]. Linking a bulk-tissue prognostic signal to both microenvironmental features and its cellular distribution may therefore provide a more informative biological framework than evaluating isolated genes.

Gene-expression studies have identified molecular subtypes and multigene prognostic signatures in EAC [8,9,10]. These reports established the potential of transcriptomic stratification, but the selected genes and reported performance vary across studies. This inconsistency is relevant in EAC, where available cohorts are generally small and apparent performance can be inflated when feature selection and evaluation are conducted in the same dataset. Appropriate resampling and evaluation in independent cohorts are therefore needed to distinguish a reproducible signal from derivation-cohort optimism [11]. External assessment should also examine biological concordance because platform, treatment, and cohort differences may limit direct transport of a survival model even when the score captures a related disease phenotype.

In this study, we restricted the TCGA esophageal carcinoma dataset to histologically confirmed EAC and sought to identify a compact transcriptional score linked to the tumor microenvironment and overall survival. Differential expression analysis, ESTIMATE, and weighted gene co-expression network analysis were used to define microenvironment-associated gene programs, followed by LASSO-penalized Cox regression to derive a four-gene score. Model optimism and selection stability were examined by bootstrap resampling and repeated nested cross-validation, and the locked score was assessed in independent transcriptomic cohorts for molecular-subtype concordance and survival discrimination. CIBERSORT, single-sample gene-set enrichment analysis, and external single-nucleus RNA sequencing were then used to characterize the immune context and cellular distribution of the model genes. This design was intended to evaluate both the prognostic signal and its biological context while defining the limits of its generalizability.

## 2. Materials and Methods

### 2.1. Study Design and Data Acquisition

RNA-sequencing count data, normalized gene-expression data, and clinical annotations were obtained from The Cancer Genome Atlas esophageal carcinoma project (TCGA-ESCA) [3]. The analysis was restricted to histologically confirmed primary esophageal adenocarcinoma (EAC). Eligible tumors had matched gene-expression, overall-survival, and clinical annotations, including available pathological stage. The final development dataset comprised 46 EAC tumors and 13 adjacent normal tissues; all 46 tumors had complete survival information. Clinical characteristics, survival data, four-gene expression values, and patient-level risk scores for the 46 EAC patients are provided in Table S1. External expression datasets were obtained from the Gene Expression Omnibus (GEO) [12]. All datasets were public and de-identified, and no new patient material was collected.

### 2.2. Differential Expression Analysis

**Figure 1:**
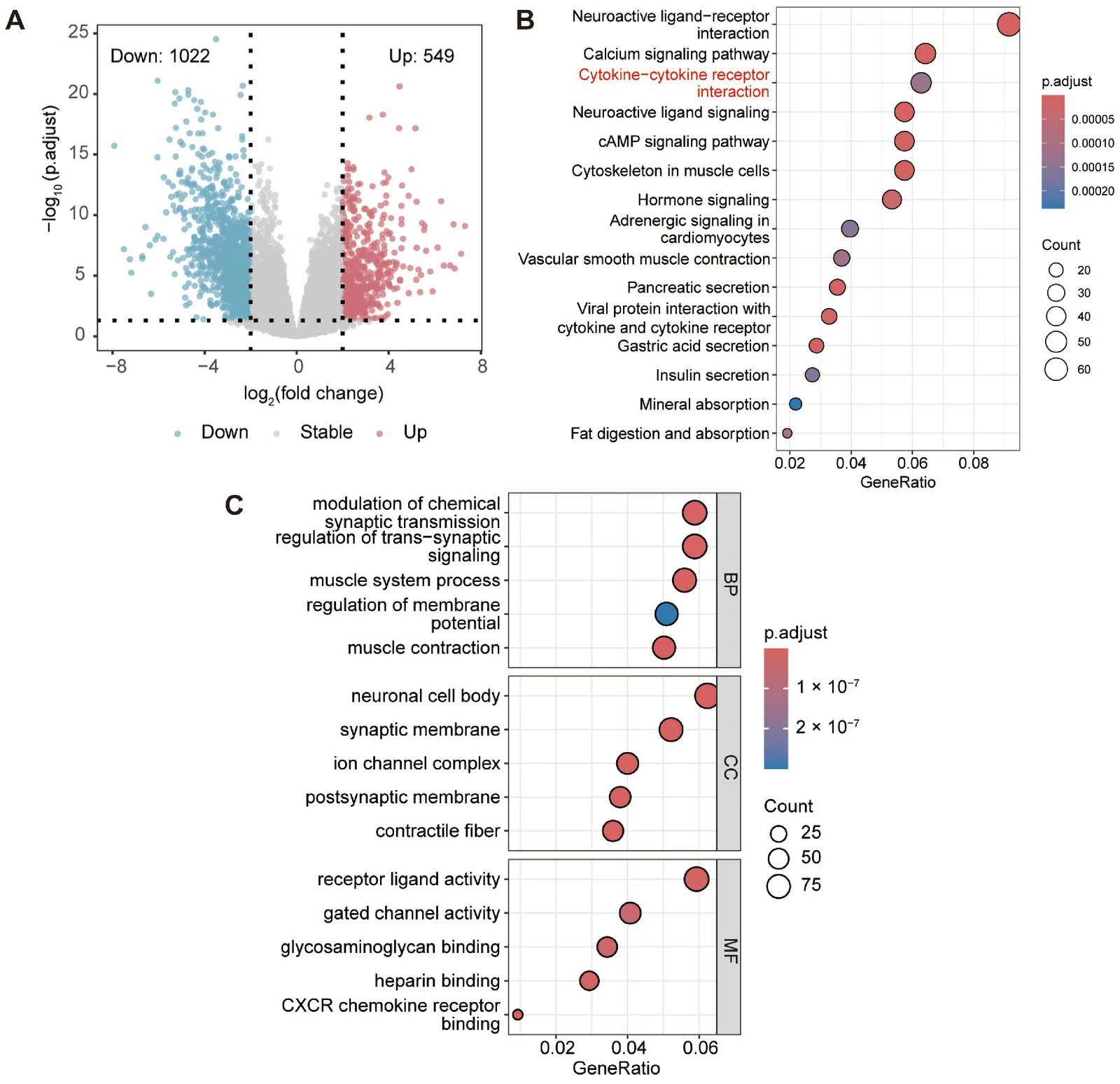
Differential expression and functional enrichment in TCGA esophageal adenocarcinoma. (**A**) Volcano plot of differentially expressed genes between 46 EAC tumors and 13 adjacent normal tissues. A total of 549 genes were upregulated and 1022 were downregulated in EAC. Vertical dotted lines indicate log_2_(fold change) = ±2; the horizontal dotted line indicates adjusted *p* = 0.05. (**B**) KEGG pathway enrichment of the differentially expressed genes. Red text highlights the cytokine–cytokine receptor interaction pathway, whose associated gene-expression heatmap is shown in Figure S1. (**C**) GO enrichment across biological process (BP), cellular component (CC), and molecular function (MF) categories. In the enrichment plots, the *x*-axis represents GeneRatio, dot size represents gene count, and color represents the adjusted *p* value.

Raw gene-level counts from the 46 EAC tumors and 13 adjacent normal tissues were analyzed with DESeq2 (v1.40.2) [13]. Genes with zero counts in every sample were removed. DESeq2 size factors were used to normalize library depth, and dispersions were estimated within the negative-binomial generalized linear model. The EAC-versus-control contrast was tested with the Wald statistic. Genes with an absolute log_2_ fold change greater than 2 and a Benjamini–Hochberg adjusted *p* value below 0.05 were defined as differentially expressed genes (DEGs; Table S2). Selected GO terms and KEGG pathways displayed in Figure 1 are provided in Table S2.

### 2.3. Functional Enrichment Analysis

To clarify the functional significance of the DEGs, Gene Ontology (GO) and Kyoto Encyclopedia of Genes and Genomes (KEGG) pathway enrichment analyses were performed with the clusterProfiler package (v4.8.3) [14]. GO annotations were subsequently grouped into three categories—biological process (BP), cellular component (CC), and molecular function (MF) [15]—and terms with a Benjamini–Hochberg adjusted *p*-value below 0.05 were deemed statistically significant.

### 2.4. ESTIMATE Scoring and Weighted Gene Co-Expression Network Analysis

StromalScore, ImmuneScore, ESTIMATEScore, and inferred TumorPurity were calculated for the 46 EAC tumors with ESTIMATE (v1.0.13) [16]. Weighted gene co-expression network analysis (WGCNA, v1.73) was then applied to the normalized expression profiles of the 1571 DEGs [17]. Samples and genes were screened with goodSamplesGenes, and sample clustering was inspected before network construction. A soft-thresholding power of 2 was selected from powers 1–20. An unsigned adjacency matrix and the corresponding topological overlap matrix were calculated, followed by average-linkage hierarchical clustering. Modules were identified by dynamic tree cutting with deepSplit = 2, pamRespectsDendro = FALSE, and a minimum module size of 30; closely related modules were merged at an eigengene dissimilarity threshold of 0.50. Gray genes were treated as unassigned.

Module eigengenes were correlated with the four ESTIMATE-derived traits using Pearson correlation. For each gene, module membership was defined as its correlation with the module eigengene, and gene significance as its correlation with the corresponding microenvironmental trait. The turquoise module showed the strongest overall associations with these traits; its 879 genes (Table S5) were therefore taken forward as the outcome-independent candidate set for survival modeling.

### 2.5. Construction of the Four-Gene Risk Score

The 879 turquoise-module genes were entered into least absolute shrinkage and selection operator (LASSO) penalized Cox regression using glmnet (v4.1-8) [18]. Overall-survival time and vital status were used as the outcome. Ten-fold cross-validation based on partial likelihood deviance selected lambda.min (lambda = 0.20). Genes with non-zero coefficients at this value formed the final model. The risk score was calculated as: risk score = (−0.4535 × *ACSM1*) + (0.0350 × *AGT*) + (0.0661 × *IGF2BP3*) + (0.4838 × *ESM1*), where each term denotes the normalized expression of the indicated gene multiplied by its locked coefficient. Patients were assigned to the high-risk group when their score exceeded the development-cohort median (1.32917), and to the low-risk group otherwise. Full-precision coefficients were retained for score calculation.

### 2.6. Evaluation in the Development Cohort

An ordinary receiver operating characteristic curve was used to describe apparent discrimination of recorded vital status in the development cohort; this was not a censoring-aware, time-dependent survival analysis. Patients were ordered by increasing risk score, and the same patient-ID sequence was used for the expression heatmap, survival-status plot, and risk-score distribution. Heatmap expression was standardized separately for each gene, and column clustering was disabled. The original, unmodified risk scores were used for the score distribution and vital-status comparison. Scores were compared between surviving and deceased patients using a two-sided Wilcoxon rank-sum test. Overall survival was estimated using the Kaplan–Meier method, with risk groups compared by the log-rank test.

### 2.7. Multivariate Cox Regression and Nomogram Construction

A multivariable Cox proportional hazards model included the continuous four-gene risk score, age, gender, overall pathological stage, T category, and N category. Hazard ratios and 95% confidence intervals were estimated with the survival package (v3.5-8). A separate nomogram was visualized using regplot (v1.1), combining the dichotomized risk group with age, gender, overall stage, T category, and N category. Because 32 and 17 patients remained at risk at 1 and 2 years, respectively, whereas only four remained at 3 years, the nomogram was limited to 1- and 2-year overall survival.

Calibration was assessed with 1000 bootstrap resamples. For each time horizon, a Cox model was fitted with cph and calibrated against Kaplan–Meier estimates using the rms calibrate function (v.8.1) (cmethod = ‘KM’, method = ‘boot’, and m = 15). Optimism-corrected observed survival probabilities were plotted against the corresponding predictions.

### 2.8. Internal Validation

Internal validation followed resampling principles for prediction models [11]. First, 1000 bootstrap samples were generated to assess the survival-modeling procedure. The 879-gene candidate set derived from the development-cohort DEG and WGCNA analyses was held fixed throughout resampling. In each sample, LASSO gene selection and penalty tuning were repeated, and performance was evaluated in both the bootstrap sample and the original cohort. The mean difference in Harrell’s C-index was subtracted from the apparent C-index to obtain an optimism-corrected estimate. Selection frequency and coefficient direction were recorded for every candidate gene.

Second, repeated nested three-fold cross-validation was performed 100 times with event-stratified folds. Gene selection and penalty tuning were confined to the inner folds, and Harrell’s C-index was calculated from predictions for patients held out in the outer folds. Both lambda.min and lambda.1se were evaluated. Finally, a simplified Cox model containing the locked score, age, and grouped stage (I–II versus III–IV) was assessed with 1000 bootstrap resamples. Its optimism-corrected C-index and calibration slope were reported. This last analysis treated the four-gene score as fixed and therefore did not correct for the preceding feature-selection step.

### 2.9. External Assessment of the Locked Score

The four coefficients were fixed before external analysis; no external gene selection or coefficient refitting was performed. Each standardized-scale weight was calculated by multiplying the original coefficient by the corresponding gene’s standard deviation in the TCGA development cohort. Within each external cohort, model-gene expression was standardized using that cohort’s mean and standard deviation without reference to outcome, and the weighted values were summed. Risk groups were assigned using the locked TCGA median on this centered scale (−0.21482), equivalent to the original development-cohort cutoff of 1.32917 after subtraction of the TCGA centering constant.

GSE13898 was used for molecular-cluster concordance [8]. The dataset contained 75 pretreatment EAC arrays from 64 patients; replicate biopsy arrays from the same patient were averaged on the log_2_ scale. When several probes mapped exactly to one model gene, their median was used. The locked score was compared across the original A, B, and C clusters with the Kruskal–Wallis test. The prespecified B-versus-A and B-versus-C comparisons used two-sided exact Wilcoxon rank-sum tests with Holm correction. Discrimination of Cluster B from Clusters A/C was summarized by a rank AUC with a 95% confidence interval from 2000 bootstrap resamples. The association between Cluster B and the locked risk group was tested with Fisher’s exact test. Although survival analyses were reported in the original study, the GEO files used here did not provide a patient-linked table containing both overall-survival time and event status. GSE13898 was therefore used for molecular-subtype concordance rather than survival validation.

GSE72873 provided 48 EAC tumor arrays, of which 44 patients had complete overall-survival information (17 deaths) [19]. For GSE254942, the deposited raw-count matrix and the clinical supplement were used [20]. Genes with zero counts across all 106 samples were removed. The remaining counts underwent DESeq2 size-factor normalization and variance-stabilizing transformation using an intercept-only design and blind = TRUE, without reference to survival outcomes [13]. No additional logarithmic transformation or batch-removal procedure was applied. Scoring was restricted to 53 patients explicitly annotated as EAC and having complete overall-survival information (22 deaths). These patients had heterogeneous recorded treatment regimens. In both cohorts, the continuous score was analyzed per one-standard-deviation increase using Cox regression and Harrell’s C-index. Inverse-probability-of-censoring-weighted time-dependent AUCs at 1, 2, and 3 years were calculated using timeROC (v0.4) [21]. The two survival cohorts were also combined in a Cox model stratified by cohort, allowing separate baseline hazards. Pooled time-dependent AUCs after within-cohort score standardization were considered secondary.

Kaplan–Meier curves were compared using two-sided log-rank tests. The locked TCGA development-cohort cutoff was used for the primary grouped analysis. Cohort-specific median cutoffs were examined only as sensitivity analyses.

Expression of the four model genes was compared between the locked risk groups using within-cohort standardized values. Mean differences were calculated as high-risk minus low-risk expression. Their 95% confidence intervals were obtained from 5000 bootstrap repetitions, resampling patients separately within each risk group. Two-sided Wilcoxon rank-sum tests were performed, with BH correction across the four genes separately within each cohort. Because the same genes contributed to risk-group assignment, these comparisons were descriptive rather than independent validation. Patient-level external scores, survival assessments, and cross-cohort expression comparisons are provided in Table S6.

### 2.10. Immune-Cell Profiling

Relative fractions of 22 immune-cell populations were estimated from the normalized tumor expression matrix with CIBERSORT (v1.03) and the LM22 signature matrix [22]. The analysis used 50 permutations, and quantile normalization was disabled because the input was RNA-sequencing data. All 46 EAC tumors were retained for risk-group comparisons. The CIBERSORT permutation *p* values were retained as model-fit diagnostics but were not used to exclude samples. Sample-level cell fractions and all 22 risk-group comparisons, including unadjusted and BH-adjusted *p* values, are provided in Table S3.

Single-sample gene-set enrichment analysis was performed with the GSVA package (v1.48.3) using curated marker sets for 28 immune-cell populations (method = ‘ssgsea’, kcdf = ‘Gaussian’, and abs.ranking = TRUE) [23]. Sample-level ssGSEA scores and all 28 risk-group comparisons, including unadjusted and BH-adjusted *p* values, are provided in Table S4. CIBERSORT fractions and ssGSEA scores were compared between the 23 high-risk and 23 low-risk tumors with two-sided Wilcoxon rank-sum tests. BH correction was applied separately to the 22 CIBERSORT comparisons and the 28 ssGSEA comparisons. Both unadjusted and BH-adjusted *p* values were reported. Unadjusted *p* values described exploratory associations, whereas statistical significance was assessed using BH-adjusted *p* < 0.05.

### 2.11. Single-Nucleus Transcriptomic Analysis

Cell-compartment localization of the four model genes was examined in GSE316062, an external single-nucleus RNA-sequencing cohort of nine EAC tumors [7]. The authors’ integrated Seurat object and curated subcompartment annotations were analyzed using Seurat (v5.1.0), without reclustering [24]. The source object contained 16,129 nuclei. The Harmony-corrected UMAP coordinates supplied in the object were used to display malignant cells, fibroblasts, basal keratinocytes, lymphocytes, myeloid cells, endothelial cells, and unassigned nuclei.

For the gene-level dot plot, the two nuclei without an assigned subcompartment were removed, leaving 16,127 nuclei in six compartments. Expression was extracted from the normalized RNA assay data layer. Dot size represented the percentage of nuclei with expression greater than zero. For color, mean log-normalized expression was calculated within each compartment, standardized within each gene across the six compartments, and truncated to the range −2.5 to 2.5 for visualization. No cell-level hypothesis tests were performed because nuclei from the same patient were not independent biological replicates.

### 2.12. Statistical Analysis

All analyses were performed in R (4.3.1), and tests were two-sided unless otherwise stated. Statistical significance was assessed using *p* < 0.05 for analyses without multiplicity adjustment and adjusted *p* < 0.05 for multiplicity-controlled analyses. BH adjustment was applied to differential expression, functional enrichment, immune-cell comparisons, and four-gene expression comparisons as specified above. Holm adjustment was used for the two prespecified GSE13898 pairwise comparisons. Unadjusted *p* values from immune-cell comparisons were interpreted descriptively. Continuous associations were summarized with Pearson or Spearman correlation as specified above. Cox models used the Efron approximation for ties. Effect estimates are presented with 95% confidence intervals where applicable. Random seeds were fixed for all resampling analyses.

## 3. Results

### 3.1. EAC Exhibits Broad Transcriptomic Alterations Involving Cytokine Signaling

To define EAC-specific transcriptional alterations, we analyzed the final development cohort comprising 46 histologically confirmed primary EAC tumors and 13 adjacent normal tissues. Differential expression analysis identified 1571 genes, including 549 upregulated and 1022 downregulated genes in EAC (Figure 1A; Table S2). KEGG analysis identified enrichment in neuroactive ligand–receptor interaction, calcium signaling, cytokine–cytokine receptor interaction, cAMP signaling, and muscle-related cytoskeletal pathways (Figure 1B). GO terms were mainly related to trans-synaptic signaling, muscle system processes, membrane-potential regulation, receptor–ligand activity, and ion-channel activity (Figure 1C). Thus, the transcriptional differences were not restricted to immune processes but involved several signaling and tissue-function programs. Cytokine–cytokine receptor interaction nevertheless represented a prominent immune-related component. The corresponding heatmap showed group-associated but heterogeneous expression patterns, with multiple chemokine and interleukin genes displaying higher expression in EAC, whereas a subset of growth factor and receptor genes was relatively enriched in normal tissues (Figure S1). These findings provided the basis for subsequent network-level identification of immune-associated gene modules.

### 3.2. WGCNA Identifies a Tumor Microenvironment-Associated Gene Module

**Figure 2:**
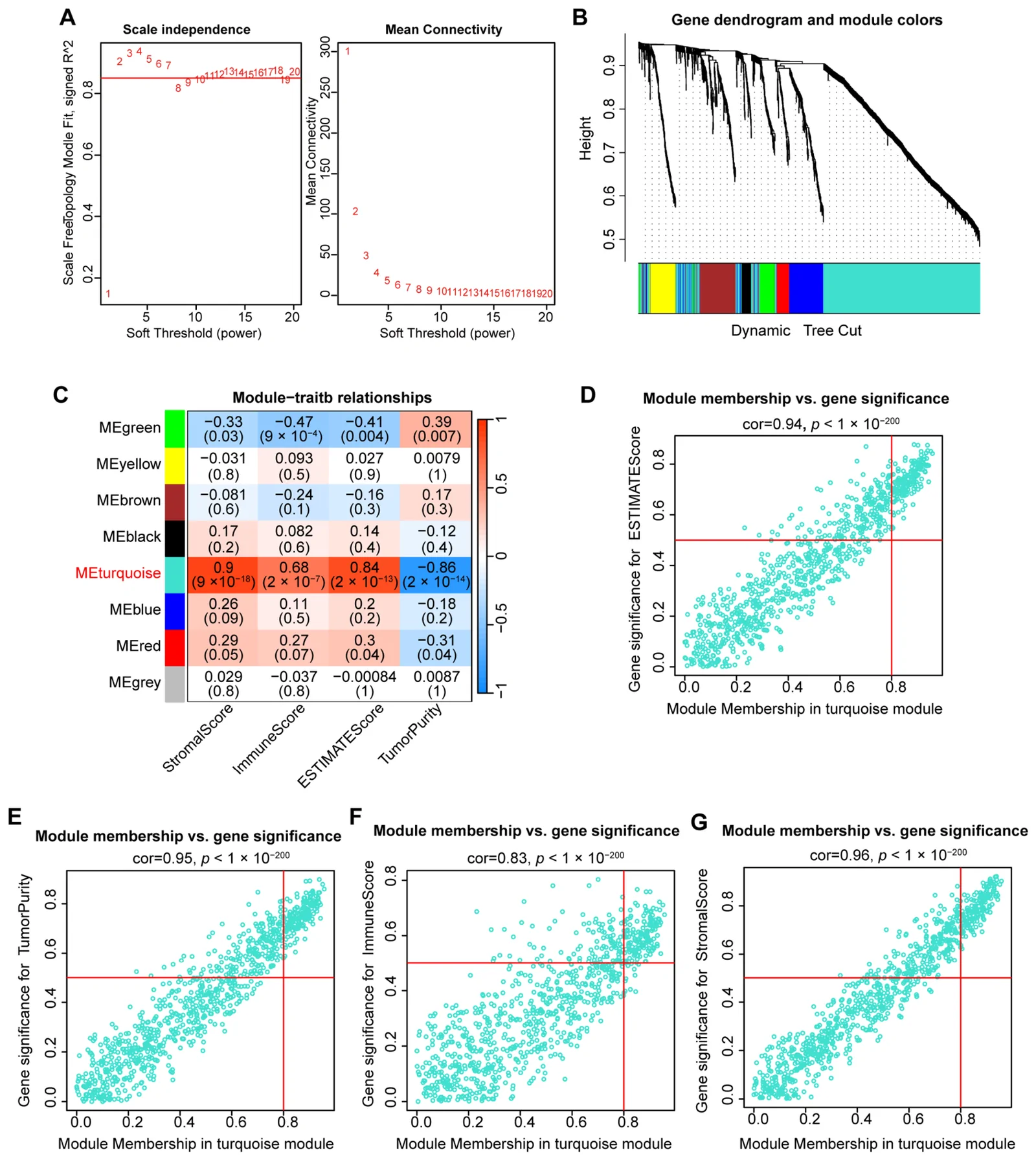
Identification of a tumor microenvironment-associated co-expression module by WGCNA. (**A**) Scale-free topology fit and mean connectivity across candidate soft-thresholding powers. A power of 2 was selected for network construction. (**B**) Hierarchical clustering dendrogram of the differentially expressed genes, with module assignments determined by dynamic tree cutting. (**C**) Pearson correlations between module eigengenes and ESTIMATE-derived traits. Correlation coefficients and corresponding *p* values are shown in each cell. (**D**–**G**) Relationships between module membership and gene significance in the turquoise module for ESTIMATEScore (**D**), TumorPurity (**E**), ImmuneScore (**F**), and StromalScore (**G**). Red lines indicate the predefined module-membership and gene-significance thresholds.

To determine whether the DEGs identified above formed coordinated tumor microenvironment-related programs, WGCNA was performed using the 1571 DEGs across the 46 EAC tumors. A soft-thresholding power of 2 was selected to approximate scale-free network topology while retaining sufficient mean connectivity (Figure 2A). Hierarchical clustering and dynamic tree cutting identified seven co-expression modules, excluding the unassigned gray genes (Figure 2B). Among these modules, the turquoise module contained 879 genes (Table S5) and showed the strongest overall associations with the ESTIMATE-derived traits. Its module eigengene was positively correlated with StromalScore (*r* = 0.90, *p* = 9 × 10^−18^), ESTIMATEScore (*r* = 0.84, *p* = 2 × 10^−13^), and ImmuneScore (*r* = 0.68, *p* = 2 × 10^−7^), and inversely correlated with TumorPurity (*r* = −0.86, *p* = 2 × 10^−14^; Figure 2C). Within the turquoise module, module membership was strongly correlated with gene significance for ESTIMATEScore (*r* = 0.94), TumorPurity (*r* = 0.95), ImmuneScore (*r* = 0.83), and StromalScore (*r* = 0.96; all *p* < 1 × 10^−200^; Figure 2D–G). These consistent associations identified the turquoise module as the principal tumor microenvironment-associated module, and its genes were subsequently considered for prognostic model development.

### 3.3. A Four-Gene Signature Stratifies Survival in the TCGA-EAC Development Cohort

**Figure 3:**
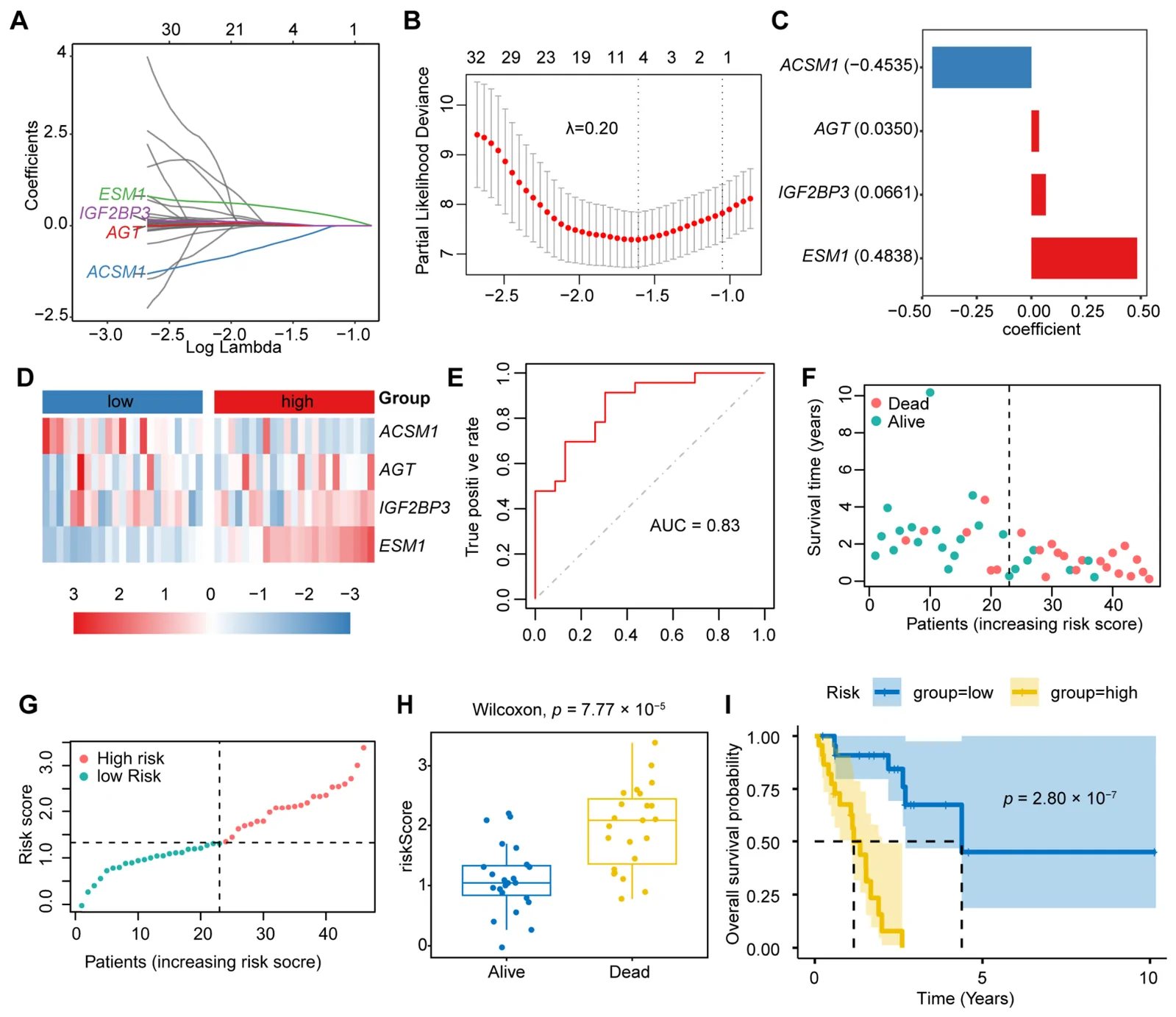
Development and apparent performance of the four-gene score in TCGA-EAC. (**A**) LASSO–Cox coefficient trajectories. (**B**) Ten-fold cross-validation based on partial likelihood deviance; dotted lines indicate λ_min_ and λ_1se_. (**C**) Locked four-gene coefficients; blue and red bars indicate negative and positive coefficients, respectively. (**D**) Gene-wise Z-score expression heatmap. (**E**) Apparent ROC curve for recorded vital-status discrimination; the diagonal dashed line indicates chance-level discrimination. (**F**) Follow-up time and vital status. (**G**) Original risk scores, with a median cutoff of 1.32917. Vertical dashed lines in (**F**,**G**) separate the low- and high-risk groups; the horizontal dashed line in (**G**) indicates the median risk-score cutoff. Panels (**D**,**F**,**G**) use the same increasing risk-score patient order, without heatmap column clustering. (**H**) Original scores by vital status, compared using a two-sided Wilcoxon test. Boxes show medians and interquartile ranges; whiskers extend to observations within 1.5 times the interquartile range. (**I**) Kaplan–Meier overall-survival curves with 95% confidence intervals; tick marks indicate censoring. The horizontal dashed line marks a survival probability of 0.50, and vertical dashed lines indicate the estimated median survival times. Each risk group contains 23 patients.

Candidate genes derived from the turquoise module were entered into LASSO-penalized Cox regression. Ten-fold cross-validation selected a penalty parameter of λ = 0.20 at the minimum partial likelihood deviance, yielding four genes with non-zero coefficients: *ACSM1*, *AGT*, *IGF2BP3*, and *ESM1* (Figure 3A–C). The resulting risk score was calculated as follows: risk score = −0.4535 × *ACSM1* + 0.0350 × *AGT* + 0.0661 × *IGF2BP3* + 0.4838 × *ESM1*. Thus, *ACSM1* contributed a negative coefficient, whereas *AGT*, *IGF2BP3*, and *ESM1* contributed positive coefficients. The four genes showed heterogeneous and overlapping expression patterns across individual tumors, and no single gene clearly separated the low- and high-risk groups (Figure 3D). Risk-group assignment therefore reflects the combined weighted contribution of the four genes rather than uniform expression changes in every model gene.

Using the development-cohort median score, the 46 patients were divided equally into low- and high-risk groups. The score showed an apparent AUC of 0.83 for distinguishing recorded vital status (Figure 3E). Deaths were more frequent in the high-risk group, and deceased patients had higher scores than surviving patients (two-sided Wilcoxon *p* = 7.77 × 10^−5^; Figure 3F–H). Kaplan–Meier analysis showed shorter overall survival in the high-risk group (log-rank *p* = 2.80 × 10^−7^; Figure 3I). These findings described apparent separation within the development cohort and were subsequently evaluated through resampling and external analyses.

### 3.4. Multivariable Analysis and Short-Term Nomogram Development

**Figure 4:**
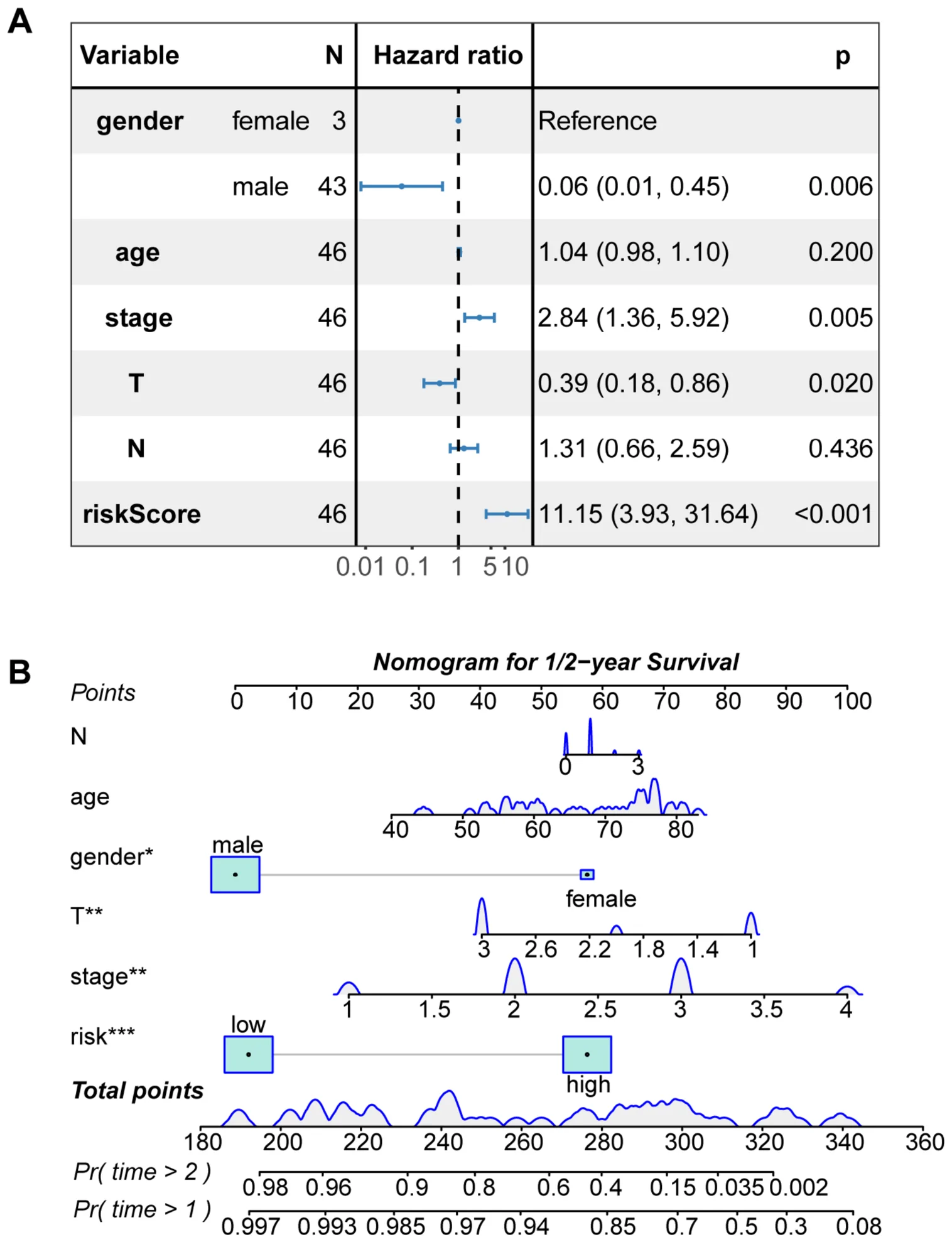
Multivariable prognostic analysis and short-term nomogram development. (**A**) Multivariable Cox regression forest plot showing hazard ratios and 95% confidence intervals for the continuous four-gene risk score and clinicopathological variables. Blue points and horizontal lines represent hazard ratios and 95% confidence intervals, respectively; the vertical dashed line indicates a hazard ratio of 1. (**B**) Nomogram integrating the dichotomized risk group with age, gender, overall stage, T category, and N category to estimate 1- and 2-year overall-survival probabilities. Blue density curves and shaded boxes summarize predictor distributions. Asterisks indicate coefficient significance in the nomogram model (* *p* < 0.05; ** *p* < 0.01; *** *p* < 0.001).

Given the survival separation observed in the development cohort, we next examined whether the four-gene risk score retained prognostic information after adjustment for clinicopathological variables. In a multivariable Cox model including gender, age, overall stage, T category, and N category, a higher continuous risk score remained associated with shorter overall survival (HR = 11.15, 95% CI 3.93–31.64, *p* < 0.001; Figure 4A). Overall stage was also associated with survival (HR = 2.84, 95% CI 1.36–5.92, *p* = 0.005), whereas age and N category were not significant. Gender and T category showed nominal associations, although these estimates should be interpreted cautiously because only three female patients were included and the anatomical staging variables were entered simultaneously.

A nomogram was subsequently constructed by integrating the dichotomized four-gene risk group with age, gender, overall stage, T category, and N category to estimate 1- and 2-year OS probabilities (Figure 4B). Bootstrap optimism-corrected calibration with 1000 resamples showed approximate agreement between the predicted and observed probabilities, with closer agreement at 2 years and greater deviation in the lower predicted-survival range at 1 year (Figure S2). Three-year predictions were not reported because only four patients remained at risk at 3 years, compared with 32 and 17 patients at 1 and 2 years, respectively. The limited late follow-up precluded reliable 3-year calibration; therefore, the nomogram was restricted to short-term prediction and should be considered exploratory.

### 3.5. Internal and External Evaluation of the Four-Gene Risk Score

Given the small development cohort, we evaluated model optimism and selection stability using complementary resampling procedures. Across 1000 bootstrap repetitions of LASSO gene selection and penalty tuning, the apparent C-index decreased from 0.837 to 0.769 after optimism correction. *ESM1* showed the highest selection frequency (71.1%), followed by *ACSM1* (32.0%). *IGF2BP3* and *AGT* were retained in 12.2% and 5.3% of bootstrap samples, respectively (Figure S3B). Repeated nested three-fold cross-validation yielded a median out-of-fold C-index of 0.589 (IQR, 0.545–0.636) for lambda.min and 0.509 (IQR, 0.500–0.540) for lambda.1se (Figure S3A). These results indicated unequal gene-selection stability and modest out-of-fold discrimination. Separately, bootstrap assessment of the simplified clinical model incorporating the fixed risk score, age, and stage yielded an optimism-corrected C-index of 0.831. Its median calibration slope was 0.858 (95% percentile interval, 0.492–1.279; Figure S3C). This conditional clinical-model assessment did not include renewed gene selection.

We subsequently applied the locked four-gene coefficients to external datasets without feature selection or coefficient refitting. In GSE13898, the score was higher in the previously defined Cluster B than in Clusters A and C [8]. It distinguished Cluster B from the other molecular subtypes with a rank AUC of 0.923 (95% CI, 0.851–0.976; Figure S4A). All 18 Cluster B cases were assigned to the locked high-risk group, comprising 47.4% (18/38) of that group. No Cluster B cases were assigned to the locked low-risk group (0/26; Fisher’s exact *p* = 9.3 × 10^−6^; Figure S4B).

In GSE72873 and GSE254942, the HRs per 1-SD increase in the locked score were 1.14 (95% CI, 0.72–1.81) and 1.04 (95% CI, 0.69–1.55), respectively. C-indices were close to 0.50, and the combined cohort-stratified HR was 1.08 (95% CI, 0.79–1.47; Figure S4C). The 1–3-year time-dependent AUCs ranged from approximately 0.49 to 0.60 (Figure S4D; Table S6).

Kaplan–Meier analyses using the locked TCGA cutoff showed no significant survival separation in GSE72873 or GSE254942 (log-rank *p* = 0.70 and 0.58, respectively; Figure S4F,H). With cohort-specific median cutoffs, the low-risk curve in GSE72873 was numerically higher over much of follow-up, although the difference was not significant (log-rank *p* = 0.30; Figure S4E). In GSE254942, the curves crossed and showed no consistent separation (log-rank *p* = 0.91; Figure S4G).

Comparison of within-cohort standardized expression revealed recurring score-associated patterns across TCGA-EAC, GSE13898, GSE72873, and GSE254942. *ESM1* was consistently higher in the locked high-risk group and showed the largest mean expression difference among the four genes in each cohort (Figure S5A). These differences remained significant after BH correction in all four cohorts (adjusted *p* < 0.001; Figure S5B). *ACSM1* showed lower mean expression in the high-risk group across cohorts, with significant differences in TCGA-EAC and GSE254942 but not in GSE13898 or GSE72873 (Figure S5A,B; Table S6). In GSE13898, *AGT* and *IGF2BP3* were also higher in the high-risk group (adjusted *p* = 1.2 × 10^−5^ and 1.4 × 10^−5^, respectively; Figure S5B). However, *IGF2BP3* showed smaller, nonsignificant increases in the other cohorts, whereas *AGT* showed no consistent direction across all four cohorts (Figure S5A,B). Because these genes contributed directly to risk-group assignment, these comparisons described score-associated expression patterns rather than independent biological or prognostic validation. Overall, the score showed molecular-subtype concordance and recurring expression patterns, particularly for *ESM1*, but generalizable survival prediction was not established.

### 3.6. Immune-Cell Profiles Across Four-Gene Risk Groups

**Figure 5:**
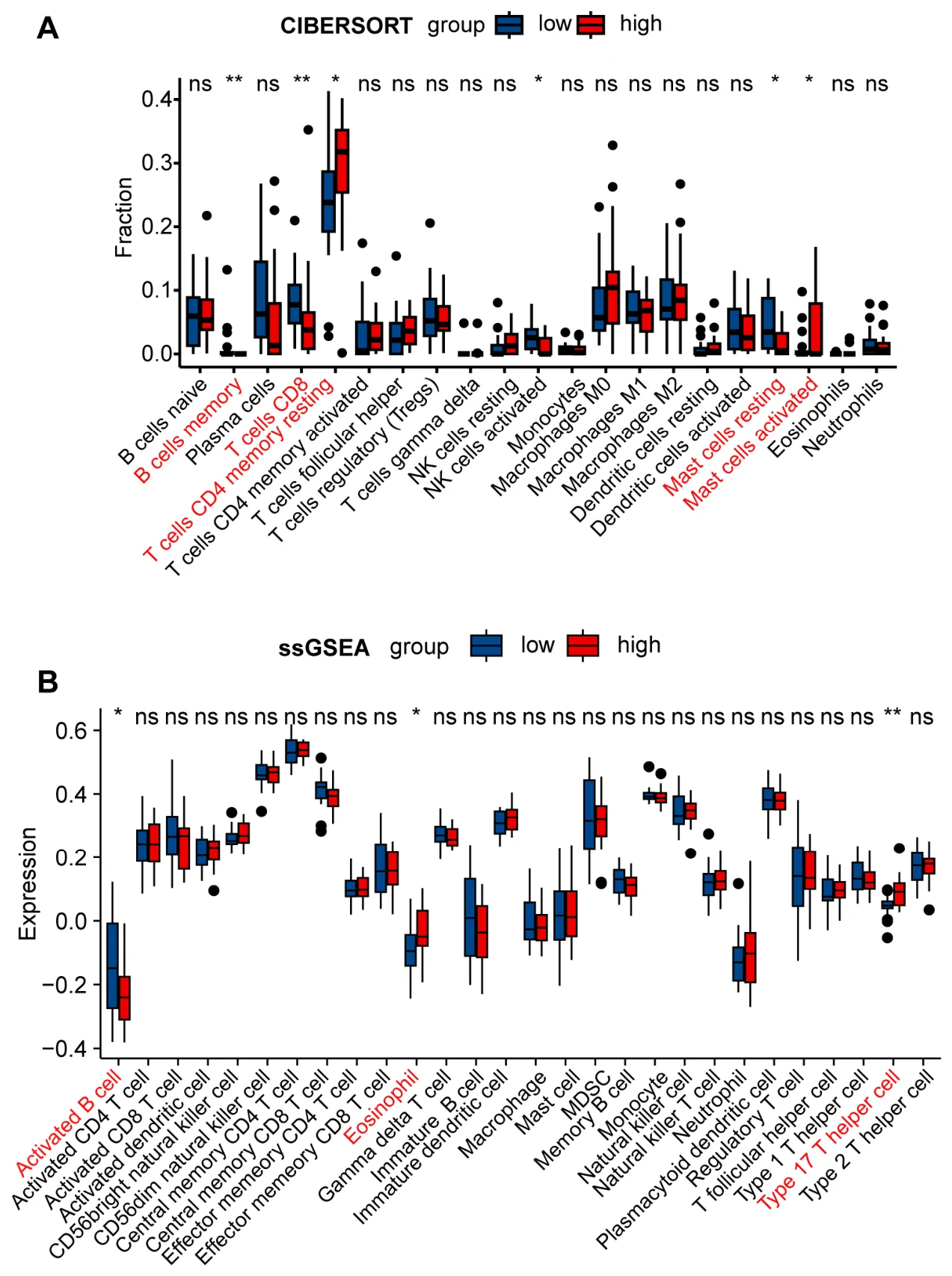
Immune-cell infiltration patterns across the four-gene risk groups. (**A**) Relative fractions of 22 immune-cell populations estimated by CIBERSORT in the low- and high-risk groups. All 46 EAC samples were included. (**B**) ssGSEA scores for 28 immune-cell populations in the two risk groups. Boxes indicate the median and interquartile range, and whiskers extend to 1.5 times the interquartile range. Group comparisons were performed using two-sided Wilcoxon rank-sum tests. Asterisks indicate unadjusted *p* values (* *p* < 0.05; ** *p* < 0.01), and ns denotes an unadjusted *p* ≥ 0.05. Red labels identify nominally significant comparisons. No comparison remained significant after BH correction within either analysis family at the 0.05 threshold.

We next explored whether the four-gene risk groups differed in estimated immune-cell composition. CIBERSORT analysis of all 46 EAC samples identified nominal between-group differences in memory B cells, CD8 T cells, resting memory CD4 T cells, activated NK cells, and resting and activated mast cells (unadjusted *p* < 0.05; Figure 5A). The high-risk group showed numerically lower median fractions of CD8 T cells, activated NK cells, and resting mast cells. Resting memory CD4 T-cell fractions were numerically higher in the high-risk group (Figure 5A).

ssGSEA also identified a limited number of nominal differences between the risk groups. Activated B-cell scores were lower in the high-risk group, whereas eosinophil and type 17 T-helper-cell scores were higher (unadjusted *p* < 0.05; Figure 5B). However, no between-group comparison remained significant after BH correction in either analysis at a false discovery rate threshold of 0.05 (Tables S3 and S4). These nominal associations were therefore considered hypothesis-generating rather than confirmatory evidence of a distinct risk-associated immune phenotype. The estimated cell fractions and enrichment scores did not directly establish functional immune suppression or T-cell exhaustion.

### 3.7. Single-Nucleus Mapping of the Four-Gene Signature

**Figure 6:**
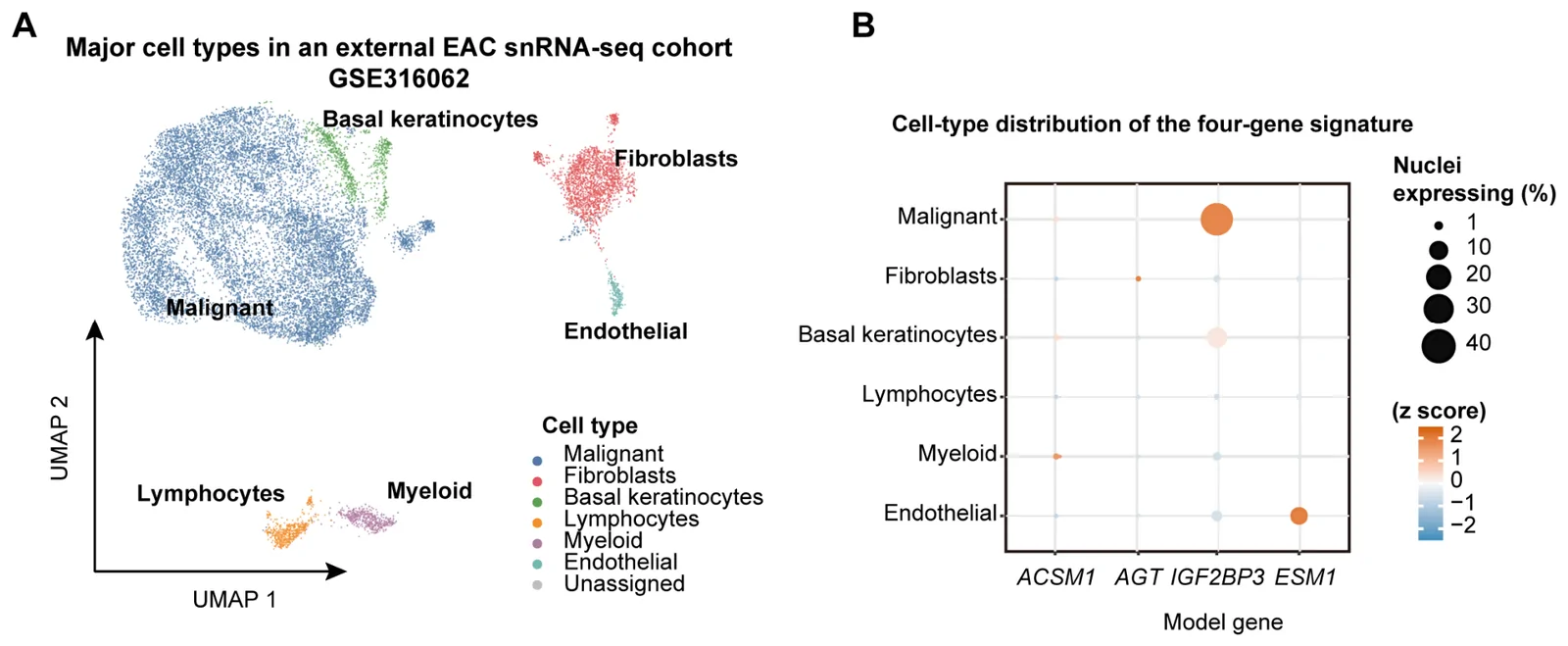
Single-nucleus localization of the four-gene signature in an external EAC cohort. (**A**) UMAP representation of the major cell compartments in GSE316062, comprising 16,129 nuclei from nine EAC patients. (**B**) Expression distribution of *ACSM1*, *AGT*, *IGF2BP3*, and *ESM1* across six assigned cell compartments. Dot size indicates the percentage of nuclei with detectable expression, and color represents the cell-type standardized mean log-normalized expression for each gene. Two nuclei without assigned compartment annotations were excluded from the dot-plot analysis.

To obtain independent cell-compartment information for the four-gene signature, we analyzed the external EAC single-nucleus RNA-sequencing dataset GSE316062. The supplied UMAP displayed 16,129 nuclei from nine patients (Figure 6A). After excluding two unassigned nuclei, 16,127 nuclei were retained across six annotated compartments for expression summaries (Figure 6B). Malignant cells comprised the largest compartment, followed by fibroblasts and basal keratinocytes.

The four genes showed distinct detection patterns across these compartments (Figure 6B). *IGF2BP3* was most strongly represented in malignant cells, where it was detected in 35.1% of nuclei, and was also detected in 11.8% of basal keratinocytes. *ESM1* showed preferential endothelial localization and was detected in 8.0% of endothelial nuclei. By contrast, *ACSM1* and *AGT* were detected in fewer than 0.25% of nuclei in every compartment, preventing reliable cell-type localization. These observations provide external cell-compartment context for *IGF2BP3* and *ESM1* but do not constitute functional or prognostic validation of the four-gene model.

## 4. Discussion

This study identified a tumor microenvironment-associated transcriptional program in EAC and derived a four-gene score comprising *ACSM1*, *AGT*, *IGF2BP3*, and *ESM1*. The score separated overall survival in the TCGA development cohort and remained associated with outcome after adjustment for clinicopathological variables. Complementary analyses provided molecular-subtype and cellular context for the score. The locked score showed concordance with the previously defined Cluster B subtype, whereas single-nucleus data localized *IGF2BP3* mainly to malignant cells and *ESM1* to endothelial cells. *ESM1* also showed recurring expression differences between the locked risk groups across four cohorts. However, immune-cell comparisons remained exploratory because none survived BH correction. These findings support investigation of score-associated transcriptional programs without establishing a reproducible immune phenotype or generalizable survival prediction.

The histology-specific and multiscale design is a key strength. EAC and esophageal squamous cell carcinoma have distinct molecular architectures, supporting their separate analysis [3]. Earlier transcriptomic studies showed that EAC contains prognostically relevant expression subtypes and gene signatures [8,25], but the present workflow first anchored candidate genes to a co-expression module strongly related to stromal, immune, and tumor-purity traits. The strong correspondence with GSE13898 Cluster B is biologically informative because that subtype was originally associated with poor outcome and coordinated expression changes rather than a single marker [8]. The single-nucleus findings add cellular context by localizing *IGF2BP3* mainly to malignant cells and *ESM1* to endothelial cells. Low detection of *ACSM1* and *AGT* prevented reliable localization of these two genes. This multicompartment view accords with recent EAC single-nucleus and spatial analyses showing that malignant-cell programs interact with endothelial, stromal, and immune states within the same tumors [7].

The immune analyses provided exploratory context for the score, although none of the between-group comparisons remained significant after BH correction. Multi-omic profiling of EAC has identified immune-suppressed, myeloid-enriched tumors with poorer survival and immune-hot tumors enriched for *CD8A*- and *NKG7*-associated signals [26]. In large tissue cohorts, greater mast-cell and NK-cell infiltration was associated with less extensive disease and longer survival [27], while coordinated T-, NK-, and B-cell infiltration carried additional prognostic information in esophageal and gastric adenocarcinoma [28]. Detailed phenotyping has also shown that EAC is immunologically heterogeneous and can contain fewer proliferating CD8 T cells and more suppressive myeloid populations than adjacent gastroesophageal tumor locations [29]. Against this background, the nominal differences observed here identify candidate immune populations for further investigation. They do not establish reduced antitumor immunity, immune dysfunction, or treatment response. The malignant localization of *IGF2BP3* and endothelial localization of *ESM1* provide more specific entry points for spatial, protein-level, and functional studies. Such studies could examine whether these cellular programs are related to the exploratory immune patterns observed in bulk tissue.

Several limitations define the scope of these conclusions. The development cohort contained 46 tumors and few outcome events, included only three women, and had limited follow-up beyond 2 years; consequently, the multivariable hazard ratio and short-term calibration estimates are imprecise. Resampling revealed modest out-of-fold discrimination and unequal gene-selection frequencies, while survival prediction was not reproduced in GSE72873 or GSE254942. The candidate-gene set was held fixed during resampling, so upstream DEG and WGCNA selection was not reevaluated. Appropriate external validation is required before a prognostic model can be considered transportable to new populations [11]. Cross-cohort differences in platform, treatment, case mix, and follow-up may have contributed to the observed variation but cannot account for it without direct testing. In addition, immune-cell abundance was inferred from bulk expression, and no risk-group comparison remained significant after BH correction. The nine-patient single-nucleus cohort supplied cell-compartment context rather than functional or prognostic validation. Larger histology-confirmed EAC cohorts with standardized clinical annotation, prespecified score calculation, spatial profiling, and protein-level assays are needed.

In summary, the study links a compact four-gene score to an independently defined EAC transcriptomic subtype and provides cellular context for two constituent genes. Recurring *ESM1* expression differences across locked risk groups further characterize the score-associated pattern but do not constitute independent biomarker validation. These observations prioritize focused investigation of malignant and endothelial programs, while the negative external survival assessments define the current limits of prognostic interpretation.

## 5. Conclusions

A tumor microenvironment-associated four-gene score comprising *ACSM1*, *AGT*, *IGF2BP3*, and *ESM1* showed survival association in the development cohort and concordance with an independently defined transcriptomic subtype. Single-nucleus analysis localized *IGF2BP3* mainly to malignant cells and *ESM1* to endothelial cells, providing cellular context for further investigation. However, external survival prediction was not reproduced, and immune-cell comparisons did not remain significant after BH correction. The score should therefore be regarded as a hypothesis-generating molecular stratification framework, rather than an externally validated prognostic predictor. Larger independent cohorts and targeted spatial, protein-level, or functional studies are required before clinical application.

## Data Availability

All datasets analyzed in this study are publicly available. RNA-sequencing and clinical data from the TCGA-ESCA project were obtained from the National Cancer Institute Genomic Data Commons portal (https://portal.gdc.cancer.gov/, accessed on 7 April 2026). The external transcriptomic and single-nucleus RNA-sequencing datasets were obtained from the NCBI Gene Expression Omnibus under accession numbers GSE13898, GSE72873, GSE254942, and GSE316062 (https://www.ncbi.nlm.nih.gov/geo/, accessed on 22 September 2026). Clinical annotations, differential-expression and enrichment results, immune profiling data, candidate genes, and external assessment results are provided in Supplementary Tables S1–S6. No new primary sequencing data were generated in this study.

## Supplementary Materials

The following supporting information can be downloaded at https://www.mdpi.com/article/10.3390/cimb48101084/s1.

## Abbreviations

The following abbreviations are used in this manuscript:

ACSM1	Acyl-CoA synthetase medium-chain family member 1
AGT	Angiotensinogen
AUC	Area under the receiver operating characteristic curve
BP	Biological process
cAMP	Cyclic adenosine monophosphate
CC	Cellular component
CD4	Cluster of differentiation 4
CD8	Cluster of differentiation 8
CI	Confidence interval
CIBERSORT	Cell-type Identification By Estimating Relative Subsets Of RNA Transcripts
C-index	Concordance index
DEGs	Differentially expressed genes
DESeq2	Differential expression analysis for sequence count data 2
EAC	Esophageal adenocarcinoma
ESM1	Endothelial cell-specific molecule 1
ESTIMATE	Estimation of STromal and Immune cells in MAlignant Tumor tissues using Expression data
GEO	Gene Expression Omnibus
GO	Gene Ontology
GSE	Gene Expression Omnibus Series accession
GSVA	Gene-set variation analysis
HR	Hazard ratio
IGF2BP3	Insulin-like growth factor 2 mRNA-binding protein 3
IQR	Interquartile range
KEGG	Kyoto Encyclopedia of Genes and Genomes
KM	Kaplan–Meier
LASSO	Least absolute shrinkage and selection operator
LM22	Leukocyte gene-signature matrix for 22 immune-cell subsets
MF	Molecular function
N	Regional lymph-node category
NK	Natural killer
OS	Overall survival
RNA	Ribonucleic acid
ROC	Receiver operating characteristic
SD	Standard deviation
ssGSEA	Single-sample gene-set enrichment analysis
T	Primary-tumor category
TCGA	The Cancer Genome Atlas
TCGA-EAC	Esophageal adenocarcinoma subset of TCGA-ESCA
TCGA-ESCA	The Cancer Genome Atlas Esophageal Carcinoma project
Th17	Type 17 T-helper cell
UMAP	Uniform manifold approximation and projection
WGCNA	Weighted gene co-expression network analysis
