# Supplementary material for: Transcriptomic and Single-Nucleus Characterization of a Tumor Microenvironment-Associated Four-Gene Score in Esophageal Adenocarcinoma
Source: Curr Issues Mol Biol. 2026 Oct 20;48(10):1084. doi: 10.3390/cimb48101084 (PMC13702898; doi:10.3390/cimb48101084)
Supplement: Supplementary file 1 [file cimb-48-01084-s001.zip › cimb-4513153 Supplementary Figures.pdf]

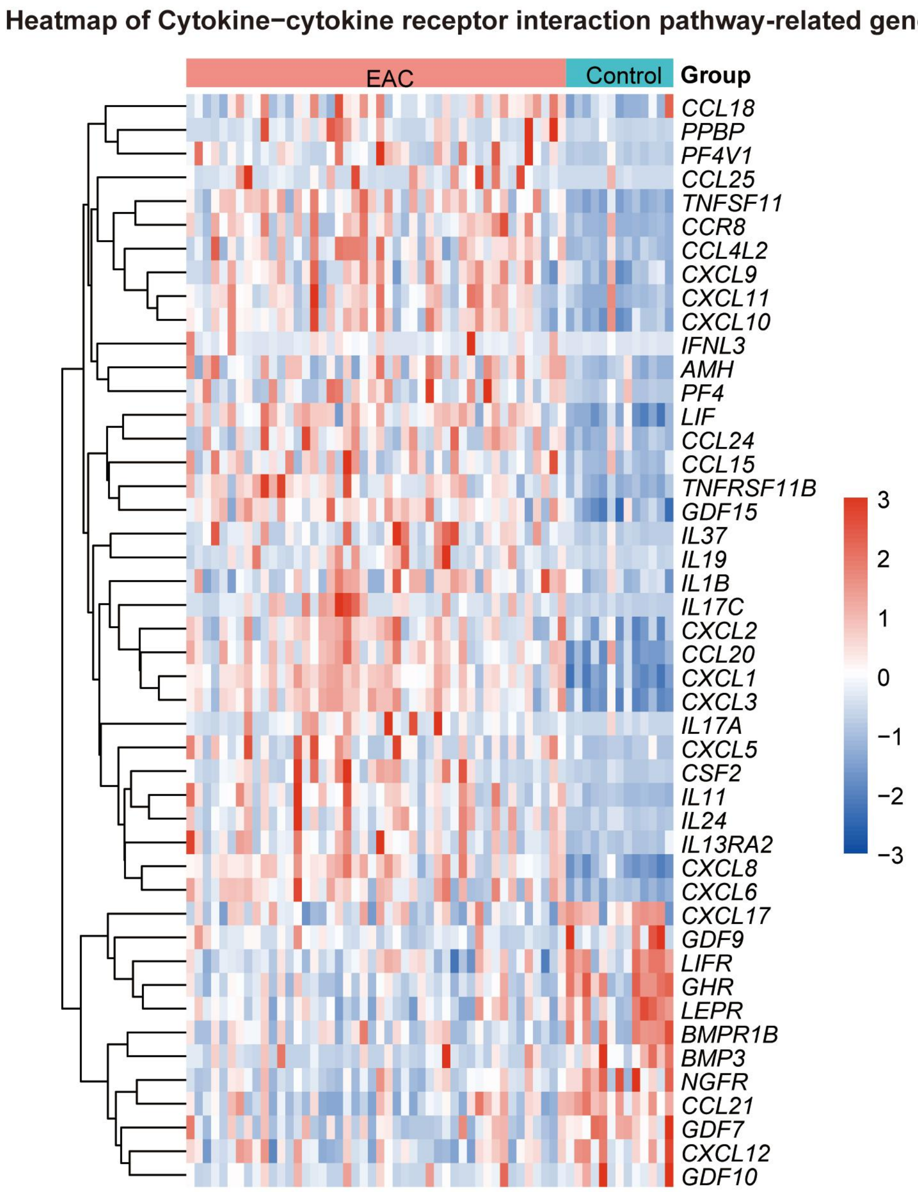

Figure S1. Expression patterns of cytokine–cytokine receptor interaction pathway genes. Heatmap showing the row-scaled expression of pathway-associated differentially expressed genes across 46 EAC tumors and 13 adjacent normal tissues. Columns represent individual samples, rows represent genes, and colors indicate gene-wise expression Z-scores.

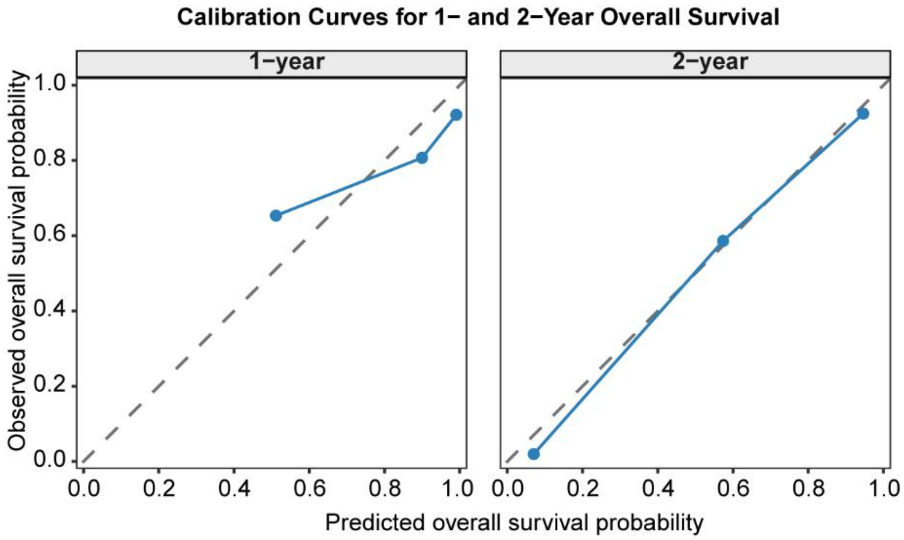

Figure S2. Bootstrap calibration of the nomogram for 1- and 2-year overall survival. Calibration curves compare the predicted and observed survival probabilities after optimism correction using 1,000 bootstrap resamples. The dashed diagonal represents perfect calibration, and the blue lines represent the optimism-corrected estimates. Three-year calibration was omitted because only four patients remained at risk at that time point.

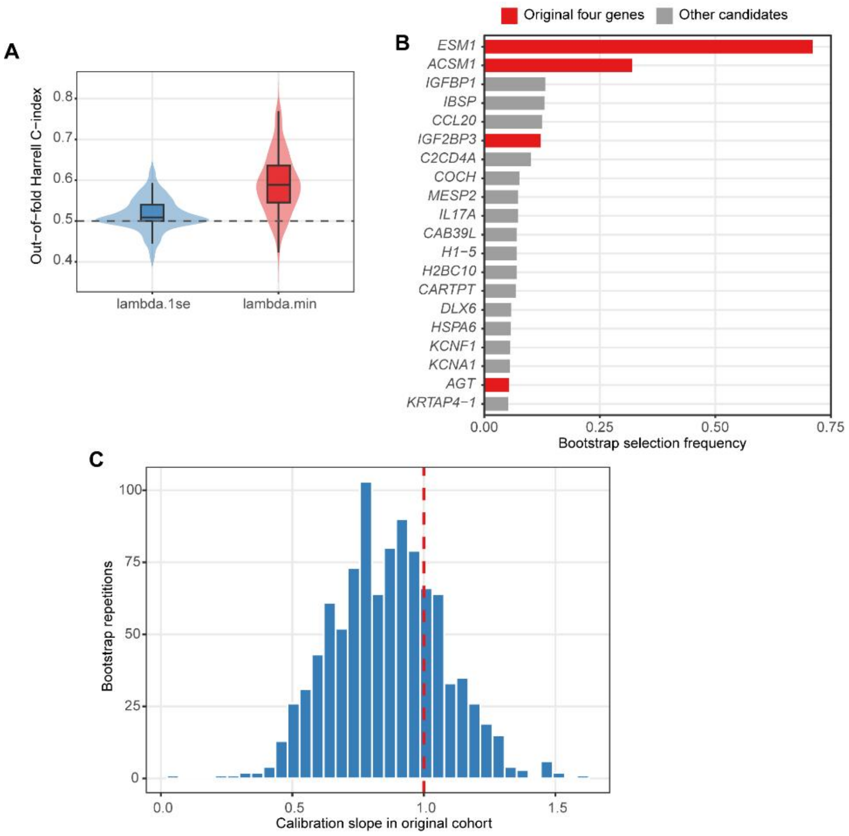

Figure S3. Internal validation and selection stability of the four-gene risk score. (A) Distribution of out-of-fold Harrell C-indices obtained from 100 repetitions of nested three-fold cross-validation using the lambda.1se and lambda.min selection rules. The dashed line indicates chance-level discrimination at 0.50. (B) Gene-selection frequencies from 1,000 bootstrap repetitions of LASSO gene selection and penalty tuning within the fixed 879-gene candidate set. (C) Bootstrap distribution of calibration slopes for the simplified Cox model containing the locked risk score, age, and stage. The red dashed line denotes the ideal slope of 1.0. Panel C represents conditional validation with the previously selected risk score treated as fixed.

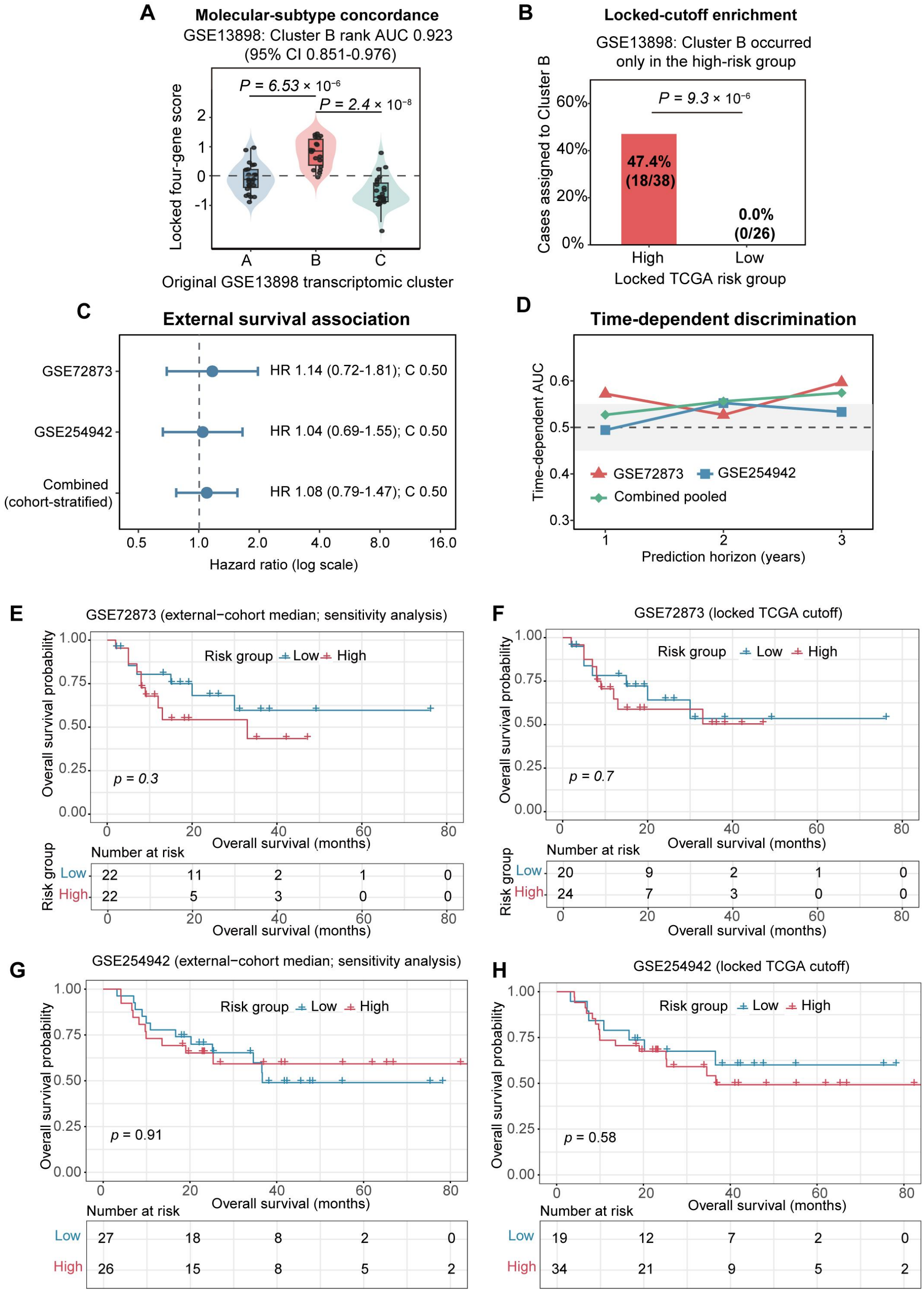

Figure S4. External evaluation of the locked four-gene risk score. (A) Score distributions across GSE13898 molecular clusters and discrimination of Cluster B. Pairwise comparisons used two-sided Wilcoxon tests with Holm correction. (B) Cluster B proportions by locked risk group, compared using Fisher's exact test. (C) Hazard ratios per 1-SD score increase with 95% confidence intervals and C-indices. The combined Cox analysis was stratified by cohort. (D) Time-dependent AUCs; pooled estimates are secondary analyses. (E–H) Kaplan–Meier curves for GSE72873 (n = 44; E,F) and GSE254942 (n = 53; G,H), using cohort-specific medians for sensitivity analyses (E,G) or the locked TCGA cutoff (F,H). Tick marks indicate censoring; numbers at risk and unadjusted log-rank P values are shown.

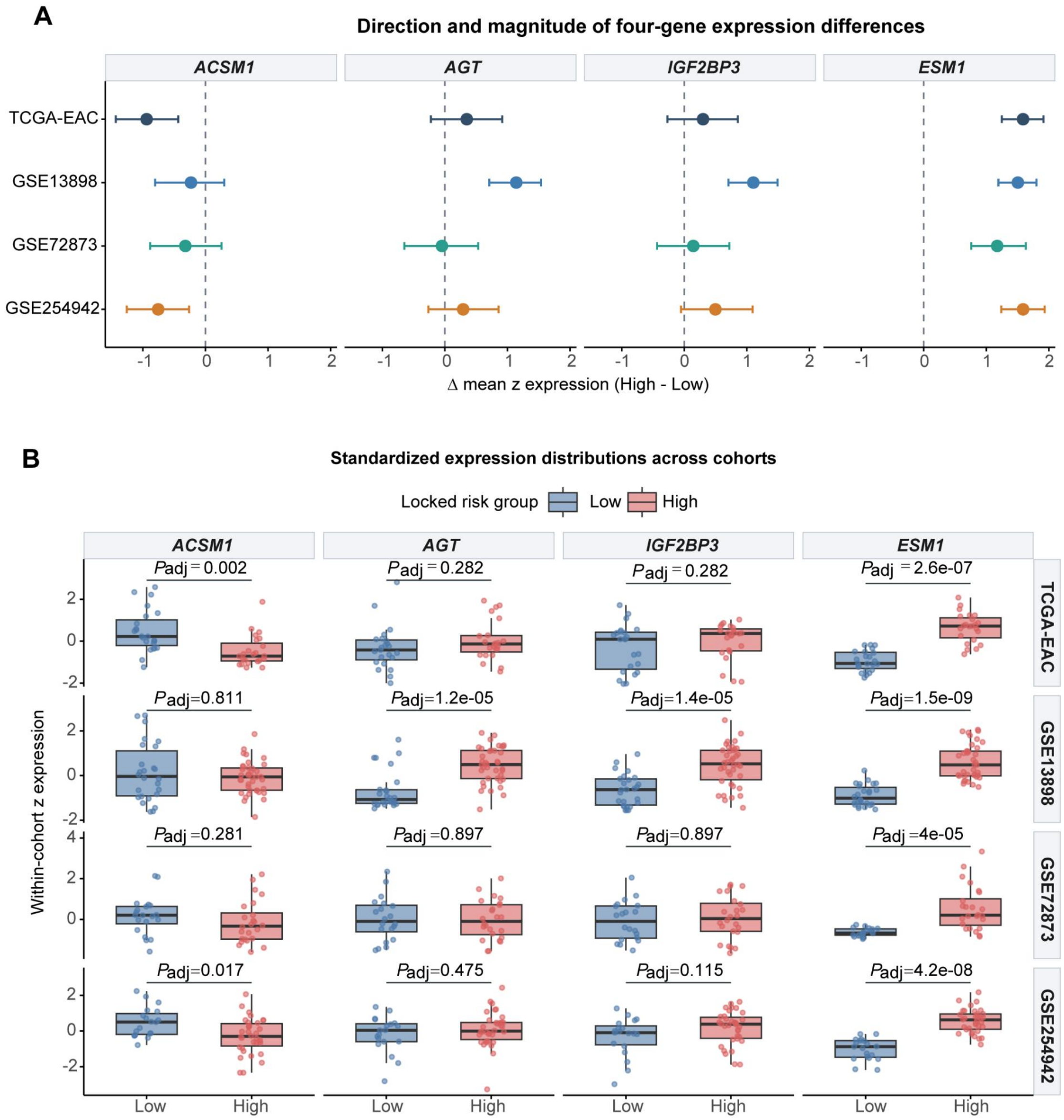

Figure S5. Score-associated four-gene expression patterns across cohorts. (A) Mean differences in within-cohort standardized expression between locked high- and low-risk groups (high minus low). Bars indicate 95% confidence intervals from 5,000 bootstrap resamples. (B) Expression distributions in TCGA-EAC (n = 46), GSE13898 (n = 64), GSE72873 (n = 44), and GSE254942 (n = 53). Boxes indicate medians and interquartile ranges; whiskers extend to observations within 1.5 times the interquartile range. Dots represent patients. Padj values reflect two-sided Wilcoxon tests with BH correction across four genes within each cohort. These comparisons are descriptive, not independent validation.
